# The Caudate Nucleus Exhibits Distinct Pathology and Cell Type-Specific Responses Across Alzheimer’s Disease

**DOI:** 10.64898/2026.01.10.694705

**Published:** 2026-01-15

**Authors:** Omar Z. Kana, Nadia Postupna, Anamika Agrawal, Brian Long, Kyle J. Travaglini, Mariano I. Gabitto, Hsin-Yu Lai, Joseph T. Mahoney, Ming Xiao, Tejas Bajwa, Heino Hulsey-Vincent, Sora L Oyaizu, Gonzalo E. Mena, Janna Hong, Emily C. Gelfand, Eitan S. Kaplan, Jeanelle Ariza, Kimberly A. Smith, Delissa A. McMillen, Samantha D. Hasting, Anish Bhaswanth Chakka, Jeff Goldy, Erica J. Melief, Zoe Juneau, Stuard Barta, Alana Oyama, Augustin Ruiz, Christina Alice Pom, Angela Ayala, Madeline Bixby, Alvin Huang, Noami Martin, Nasmil Valera Cuevas, Paul Olsen, Josh Nagra, Rushil Chakrabarty, Michael Tieu, Trangthanh Cardenas, Amy Torkelson, Darren Bertagnolli, Junitta Guzman, Rebecca Ferrer, Jack Waters, Thomas J. Grabowski, Paul K. Crane, Nicole M. Gatto, Jeremy A. Miller, Rebecca D. Hodge, Michael Hawrylycz, C. Dirk Keene, Ed S. Lein, Jennie L. Close

## Abstract

Aβ presence in the caudate nucleus (Ca) partially defines Thal stage III in Alzheimer’s disease (AD), but little is known about AD’s cellular impact on the region. Leveraging a public basal ganglia taxonomy of cellular populations, we generated a cellular resolution atlas of AD-associated pathological changes in Ca. Unlike cortex, we found that Ca AD pathology is dominated by two key features: phosphorylated tau (pTau)-containing neuropil threads enriched near oligodendrocytes in white matter tracts and amyloid-β diffuse plaques enriched in gray matter. Although AD pathology in affected cortical regions results in neuronal loss, we find no AD-driven reductions in neuron proportions in Ca. However, there were observable changes in multiple cellular populations. Protoplasmic astrocytes and FLT1+/IL1B+ microglia increased in abundance with global pTau levels. We also observe gene expression changes in fast-spiking PTHLH-PVALB interneurons indicative of disrupted signaling pathways and altered intrinsic physiological properties. This work provides a cellular-resolution framework for understanding AD pathology in Ca.

## Full Text Availability

The license terms selected by the author(s) for this preprint version do not permit archiving in PMC. The full text is available from the preprint server.

